# Structure and Electronic Properties of InSb Nanowires Grown in Flexible Polycarbonate Membranes

**DOI:** 10.3390/nano9091260

**Published:** 2019-09-05

**Authors:** Abhay Pratap Singh, Kevin Roccapriore, Zaina Algarni, Riyadh Salloom, Teresa D. Golden, U. Philipose

**Affiliations:** 1 Department of Physics, University of North Texas, Denton, TX 76203, USA (A.P.S.) (K.R.) (Z.A.); 2 Oak Ridge National Lab. (ORNL), Oak Ridge, TN 37830, USA; 3 Department of Materials Science and Engineering, University of North Texas, Denton, TX 76203, USA; 4 Department of Chemistry, University of North Texas, Denton, TX 76203, USA

**Keywords:** polycrytaline InSb nanowire, electrochemical deposition, space charge limited current (SCLC), polycorbonate template

## Abstract

A dense array of vertically aligned indium antimonide (InSb) nanowires with high aspect ratio (diameter 150 nm, length 20 μm) were grown in the pores of a track-etched polycarbonate membrane via a one-step electrochemical method. There are several reports on InSb nanowire growth in the pores of a mechanically rigid, nano-channel alumina template (NCA), where nanowire growth occurs in the pores of the NCA. This work on InSb nanowire growth in pores of track-etched polycarbonate (PC) membrane sheds light on the various factors that affect nucleation and nanowire growth. The average length and diameter of the as-grown nanowires was about 10 μm and 150 nm, respectively. Two possible mechanisms accounting for two different morphologies of the as-grown nanowires are proposed. The polycrystallinity observed in some of the nanowires is explained using the 3D ‘nucleation-coalescence’ mechanism. On the other hand, single crystal nanowires with a high density of twin defects and stacking faults grow epitaxially by a two-dimensional (2D) nucleation/growth mechanism. To assess the electrical quality of the nanowires, two- and four-terminal devices were fabricated using a single InSb nanowire contacted by two Ni electrodes. It was found that, at low bias, the ohmic current is controlled by charge diffusion from the bulk contacts. On the other hand, at high bias, the effects of space charge limited current (SCLC) are evident in the current–voltage behavior, characteristic of transport through structures with reduced electrostatic screening. A cross-over from ohmic to SCLC occurs at about 0.14 V, yielding a free carrier concentration of the order of $10^{14}$ cm${}^{-3}$.

## 1. Introduction

One-dimensional nanowires exhibit novel physical, optical and electronic properties, making them attractive for applications as interconnects and as nanoscale electronic, optoelectronic devices. Several types of nanowires including metallic, semiconducting and organic nanowires have been synthesized and of the semiconducting class of nanowires, group III–V materials like indium antimonide (InSb) show great promise as devices [1]. InSb has a direct band gap of 0.17 eV at 300 K, and high electron mobility [2,3] of 77,000 cm${}^{2}$ V${}^{-1}$s${}^{-1}$. The small electron effective mass of 0.014, [2,4] and large Lande g-factor of 51 [4,5] make it a promising material in applications such as high-speed electronic devices, low-power logic transistors [6,7], nanowire field effect transistors (FETs) [8,9,10], infrared (IR) nano-optoelectronics [11,12,13], thermoelectrics [14,15,16,17,18], and magnetoresistive sensors [19].

There are several nanowire growth techniques including high temperature growth by chemical vapor deposition (CVD) [20] and high-vacuum growth by molecular beam epitaxy (MBE) [21]. In these cases, ordered growth of dense nanowire arrays requires patterning of the substrate with seed layers, the entire process requiring very sensitive control of the growth environment, resulting in a complex, expensive and non-scalable nanowire growth technique. An extremely successful approach for growing ordered arrays of nanowires with a high aspect ratio is electrochemical growth in non-conducting porous membranes. This relatively inexpensive and versatile growth can be performed at room temperature, and is preferred for growth of compound semiconductors like InSb where the difference in vapor pressures between In and Sb can result in non-stoichiometric growth at high temperatures. The technique is especially desirable for growing heterostructures since it prevents heat-induced inter-diffusion of elements across adjacent layers in the heterostructure. Another advantage is the possibility that the nanowire can be doped during the electrodeposition process, thus making it the method of choice for synthesis of nanostructured materials at a low cost. There are several reports on growth of metallic nanowires in pores of polycarbonate membranes, whereas most semiconductor nanowires are grown in alumina templates. Thus, the first challenge is to determine an appropriate template, with the required geometry in terms of pore diameter, channel length and surface chemistry that will facilitate total removal of template after nanowire growth without compromising the surface or composition of the nanowires.

The motivation for this study is the low-temperature synthesis of nanowires in pores of a flexible template. This is of interest in fabrication of stretchable devices that leads to promising applications in wearable and futuristic technology, including biometric and optoelectronic devices. Moreover, InSb nanowire growth in polycarbonate membranes allows producing nanowires with uniform diameters and relatively smooth surfaces when compared with nanowire growth in an alumina template. Though the pore density is significantly lower than anodized alumina templates, the realization of an array of nanowires in a flexible PC membrane raises the possibility of realizing composite nanostructures with unique functionalities.

In this work, we study the efficacy of using track-etched polycarbonate (PC) membranes as a template for InSb nanowire growth. The PC membranes contain a high density of uniform cylindrical pores aligned perpendicular to the surface and penetrating the entire thickness of the template [22,23,24,25]. It therefore allows for ordered growth of nanowires, promising for applications related to energy [26,27,28] and electronic devices [29,30,31] where the array format of nanowires is preferred; the major advantages of array-based devices being device density, process scalability, reproducibility in terms of dimensions and cost effectiveness [32]. There are works on InSb nanowires grown in AAO template pores, but, to the best of our knowledge, there are no reports on InSb nanowire growth in the PC membrane. In this paper, we present our findings on the synthesis and characterization results of InSb nanowires that are electrochemically grown in template pores and we present a model explaining the role of electrodeposition parameters on the sample crystallinity.

## 2. Experimental Method

A commercial nanoporous PC track-etched membrane (Millipore Sigma, St. Louis, MO, USA), of pore length and diameter of ∼20 μm and ∼100 nm, respectively, was used for nanowire synthesis. A thin layer of gold (Au) (∼200 nm) was thermally evaporated on to one side of the membrane using an Auto 306 (HHV Technologies, Bangalore, India) thermal evaporator. The Au layer functions as a contact electrode and also as the nucleation site for nanowire growth inside the porous template. Electrodeposition of InSb was conducted in a three electrode flat cell (Model K0235, Princeton Applied Research, Oak Ridge, TN, USA) with the template functioning as the working electrode, platinum mesh (2.54 cm × 2.54 cm) as a counter electrode, and Ag/AgCl as a reference electrode. A potentiostat (Princeton Applied Research, model: 263A) was used to apply a constant potential (between −1.0 and −1.5 V) with respect to the reference electrode (Ag/AgCl) for the entire electrodeposition time period of 15 min. The value of the applied potential was decided based on data obtained from cyclic voltametry experiment discussed in the next section. The electrolyte was a solution containing 0.15 M indium chloride (InCl${}_{3}$), 0.1 M antimony chloride (SbCl${}_{3}$), 0.36 M citric acid (C${}_{6}$H${}_{8}$O${}_{7}$H${}_{2}$O), and 0.17 M potassium citrate (C${}_{6}$H${}_{5}$K${}_{3}$O${}_{7}$) and its pH value was adjusted to 1.8.

Post-growth, the PC membrane was carefully rinsed several times with DI water and subsequently placed in a clean Corning Centristar centrifuge vial (Corning, NY, USA) as part of the procedure to dissolve the template and allow nanowires to be extracted from the template pores. Using a disposable pipette, a few drops of dichlorobenzene solution were added to the vial and, after 10 min of gentle agitation, the solution was sonicated for about 5 s. The visual impression at this stage was that the PC membrane was completely dissolved. However, there are some traces of the membrane that remain in solution. The sonication process was found to be aggressive and resulted in breakage of nanowires and so its duration was kept very short. To remove traces of the membrane that stick to the nanowire surface, the solution containing nanowires was diluted with isopropyl alcohol (IPA) and centrifuged (Hermle model Z206A, Goshein, Germany) at 3000 rpm for 2 min. The residue at the bottom of the vial contains the nanowires and so this residue was repeatedly (∼4 to 5 times) treated with dichlorobenzene followed by IPA wash. The nanowires were finally placed in a vial containing IPA and its concentration in solution was adjusted by varying the volume of IPA.

To characterize the as-grown InSb nanowires, an electron microscope (JEOL JSM 7001F SEM, Peabody, MA, USA) and Hitachi SU1510 (Tokyo, Japan) equipped with energy dispersive X-ray spectroscopy (EDX) was used to study the morphology and composition of the nanowires. To study the crystalline nature of the nanowires, the nanowires were placed on a lacy carbon TEM grid and studied by transmission electron microscopy (TEM: A NION UltraSTEM 200 scanning transmission electron microscope (KirkLand, WA, USA) equipped with a 3rd generation C3/C5 aberration corrector operating at 200 kV) and Selected Area Electron Diffraction (SAED). The nanowires were also characterized by Raman spectroscopy where the spectrum was recorded at ambient temperature on a Nicolet Almega XR Dispersive Raman spectrometer (Waltham, MA, USA), using a 532 nm green laser. High resolution X-ray diffraction studies were performed using a Rigaku Ultima III X-ray diffraction (XRD) with CuKα tube (λ = 1.5406) (Tokyo, Japan). For electron transport measurements, a droplet of the solution containing the nanowires was placed on SiO${}_{2}$ (200 nm)/n${}^{+}$ Si substrate and an isolated, sufficiently long nanowire was identified under a scanning electron microscope (JEOL JSM 7001F) and its position marked with reference to pre-patterned markers. Two electrodes separated by 1.5 μm were defined by electron beam lithography (JEOL JSM 7001F w/ XEON patter writer). Prior to metal (300 nm thick Ni film) deposition, a reactive ion etch (AGS RIE MPS-150) “descum” process was used to ensure excellent metal film adhesion by removing any remaining resist not fully washed away by development. Nickel has a work function that closely matches that of InSb and thus is expected to provide good ohmic contact to the nanowire. Following lift-off of the resist, the device comprising of a single nanowire contacted by Ni electrodes was studied using an Agilent B1500A semiconductor parameter analyzer (Santa Roza, CA, USA).

## 3. Results and Discussion

InSb nanowire growth occurred under potentiostatic conditions through a multi-step reaction process involving various ions responsible for the electrochemical deposition of InSb [33,34]. With reference to Ag/AgCl electrode, the balanced reaction and the overall electrode potential for indium (In) and antimony (Sb) deposition is expressed as:

$${\mathrm{In}}^{+3}+3e^{-}\to \mathrm{In}\left(E^{0}=-0.46V\right),$$

$${\mathrm{SbO}}^{+}+2H^{+}+3e^{-}\to \mathrm{Sb}+H_{2}O\left(E^{0}=+0.39V\right).$$

**Figure 1:**
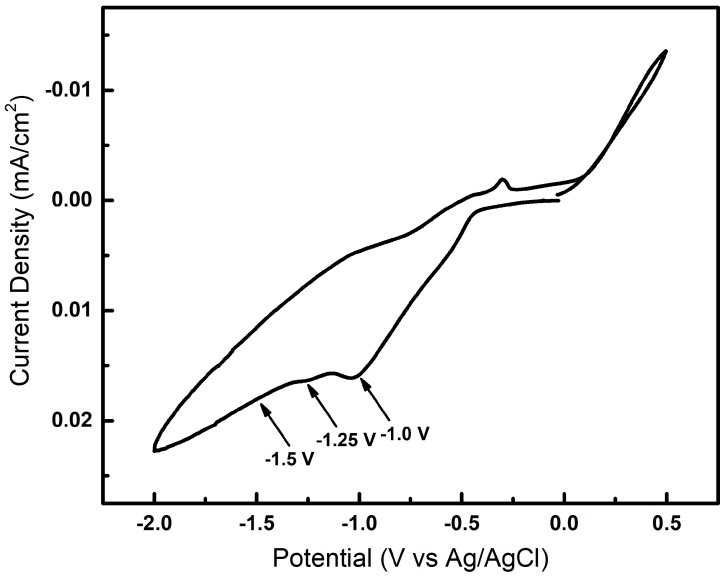
Results from cyclic voltammogram showing variation of current with positive (oxidizing) potentials and negative (reducing) potentials. Increasing the negative potential beyond −1.5 V leads to hydrogen evolution.

Prior to electrodeposition, the equilibrium deposition potential was determined using cyclic voltammetry and three different deposition potentials of −1.0. −1.25 and −1.50 V were used to grow nanowires. A reduction potential more cathodic than −0.46 V is needed to deposit the nanowires. Cyclic voltammetry (CV) was used to help determine the best deposition potential for the plating bath. Figure 1 shows the data from the CV experiment, where the potential was swept in the cathodic direction beginning at +0.0 V to −2.0 V and back to +0.5 V. There is minimal current flow until close to ≈−0.5 V, where the current increases rapidly to the final potential at −2.0 V. A small reduction peak can be seen at ≈−1.0 V corresponding to the deposition of InSb. At a more cathodic potential than −1.0 V, the current continues to increase and bubbles can be observed corresponding to hydrogen evolution at the substrate surface. To ensure a stoichiometric InSb deposition, potential values greater than −1.0 V are applied to the working electrode. The rates of reduction for both In${}^{+3}$ and Sb${}^{+3}$ ions in solution can be controlled by careful selection of the electrochemical parameters (i.e., pH, temperature, potential, concentrations) facilitating co-deposition.

It is likely that In and Sb exhibit an anomalous codepostion mechanism where the nucleation of one species affects the deposition of another species in solution. This is common in iron based alloys and also seen in other semiconductor electrodepostion reactions [35,36]. The implication of a high negative electrode potential on the stoichiometry of InSb thin films has been discussed by Ortega et al. [37]. Most InSb electrochemical depositions therefore occur at about −1.5 V. The reactions involved in the nanowire growth mechanism is most likely more complex and the quality of the growing crystal, its morphology and composition were found to be critically dependent on the pH value of the electrolyte, the applied potential as well as the type and aspect ratio of the template pores in case of template-assisted electrochemical growth. As seen in Figure 1, as the voltage is swept to −2.0 V, a reduction peak occurs around −1.1 V; the increase in cathodic current beyond this voltage results in hydrogen evolution. Based on the cyclic voltammetry data, InSb nanowire synthesis was performed under potentiostatic conditions at three different potentials: −1.0, −1.25 and −1.50 V.

**Figure 2:**
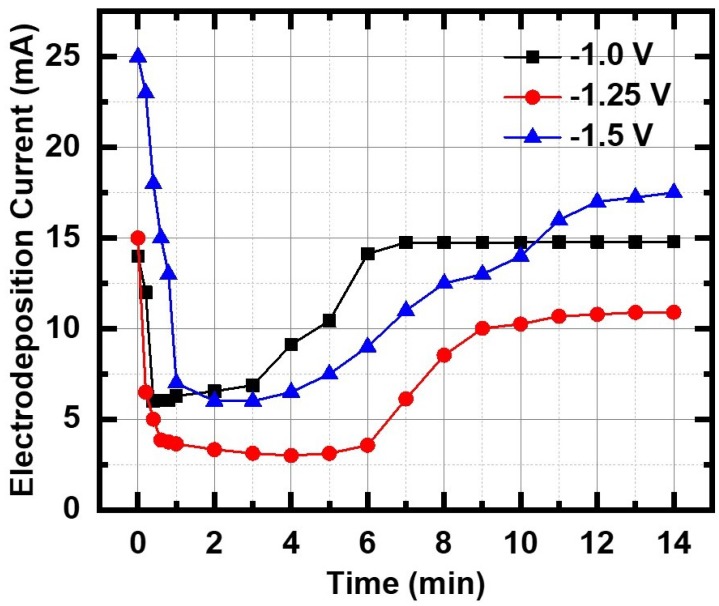
Current transient of room temperature InSb nanowire growth in Au-coated polycarbonate membrane at three different deposition potentials: −1.0 V, −1.25 and −1.50 V.

The nucleation and growth kinetics of InSb deposition on Au electrode in the PC membrane was studied using the current transient technique for the three different applied potentials (Figure 2).

The mechanism depends strongly on the InSb–Au interaction and, since the Au film was thermally evaporated on the back of the PC membrane, it is most likely an amorphous film which does not provide any epitaxial influence to the nanowire growth process, which is not a steady state process. As seen in the curves of Figure 2, the plot resembles the classical Stranski–Krastanov (S-K) (layer + island) growth model with the current–time transient showing four characteristic regions: (i) a sharp drop in the initial current density due to discharging of the double layer. The thickness of the double layer depends on the total ion concentration in the electrolyte. The decrease in current signifies the stage where nanowire growth is initiated as the In and Sb ions diffuse through the double layer to the Au electrode at the bottom of each pore, following Fick’s first law; and (ii) a constant low current region (induction period $t_{ind}$) corresponding to the nucleation time, when many nuclei with random orientation form on the Au nanostructured surface inside the pores. As seen in the plot, $t_{ind}$ varies with deposition potential and, in this particular experiment, its value was a minimum at the higher deposition potential of −1.50 V. $t_{ind}$ varies for each run, indicative of the randomness of the nucleation stage. It was also noted that, at the large negative potential of −1.5 V, there was some hydrogen evolution from the pores, a process that gets faster as the negative potential is increased; (iii) an increase in the current density corresponding to growth of columnar structure inside the pores. The magnitude of the current density and the growth period depend on the electrolyte composition, nature of the working electrode, pH of solution, deposition potential, etc.; (iv) a limiting (steady state) current, where the current magnitude is limited by diffusion of the ions to the top surface of the template [33].

**Figure 3:**
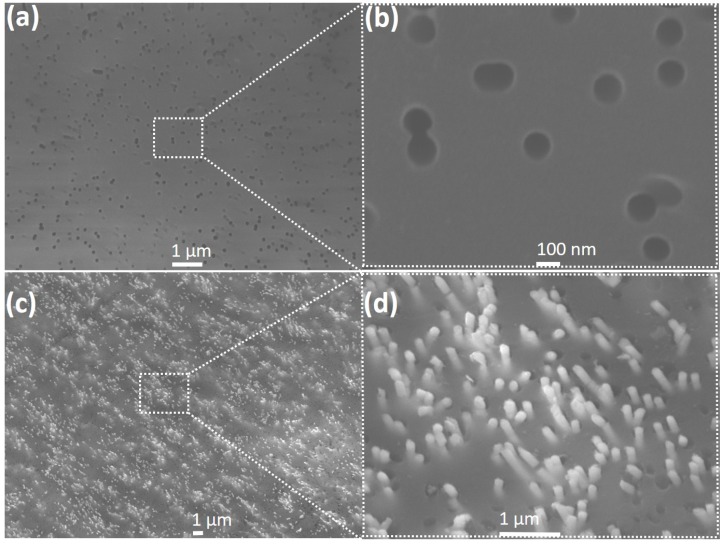
SEM images of blank polycarbonate membrane before and after growth of InSb nanowires (**a**) SEM image of bare polycarbonate template; (**b**) zoomed in SEM image of polycarbonate template with different diameters and shape of pores, ranging from ~150 – 200 nm; (**c**) SEM image of InSb NW exposed tips after 30 s of oxygen plasma etching; and (**d**) zoomed in SEM image of exposed tips of InSb NWs.

To investigate the effect of electrodeposition on the template morphology, the PC membrane was first studied to verify the pore density and pore-size. Figure 3a is an SEM image of the blank PC template, showing variable pore diameters and shapes and indicating about 20% porosity.

**Figure 4:**
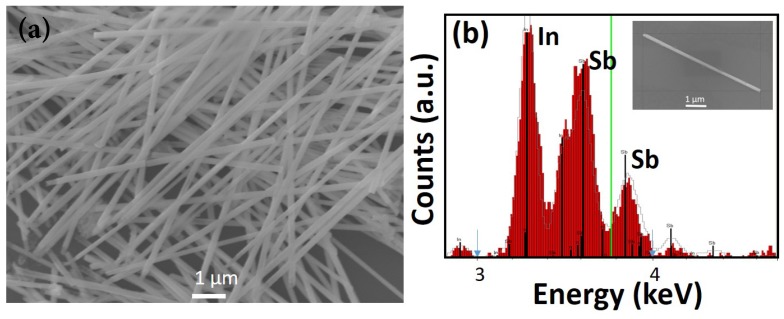
(**a**) SEM images of bundle of InSb nanowires after drop casting on silicon wafer; (**b**) Energy-dispersive X-ray (EDX) spectrum of a single and short (~5 μm) InSb nanowire that is shown in the inset of the figure (**b**).

A magnified section of the template (indicated by the square in Figure 3a) clearly shows that the pores are not uniformly spread out over the membrane surface (Figure 3b), unlike those found in alumina templates. Following InSb nanowire growth at −1.5 V, the template surface was etched with O${}_{2}$ plasma for 5 min at 100 Watts and 100 mTorr pressure and examined under the SEM. As seen in Figure 3c, nanowires grew profusely in the template pores and their tips are clearly visible. A zoomed-in version of the marked section of the membrane is shown in Figure 3d. This image showing protruding tips of the nanowires verifies that nanowire growth occurred in the template pores. The nanowires were then released into solution (following the procedure explained in the previous section); a droplet of this solution was found to contain a high density of uniform nanowires, as seen in the SEM image of Figure 4a.

The nanowires tend to clump together and efforts to separate them by sonication resulted in breakage, most likely caused by mechanical stress induced by the process. The as-grown nanowires were found to have uniform diameters but varied lengths; the average length of the nanowires was determined to be about 10 μm, comparable to the thickness of the template. The nanowires had diameters in the range of 120–150 nm, corresponding to the template pore diameter. EDX analysis of a single nanowire (inset of Figure 4b) shows a stoichiometric composition (Figure 4b).

**Figure 5:**
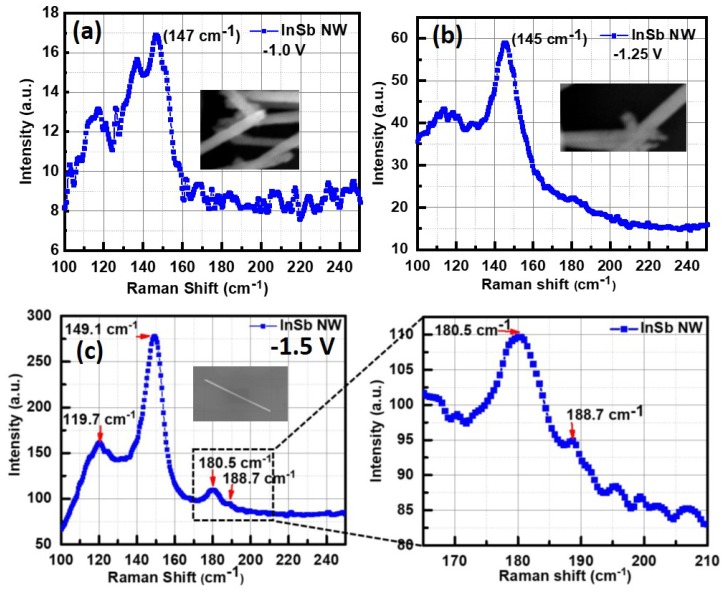
Raman spectrum of electrochemically grown InSb nanowire in porous polycarbonate template. (**a**) at a deposition potential of −1.0 V, the spectrum shows a characteristic defect related peak at 147 cm${}^{-1}$ and around cm${}^{-1}$. No peaks were observed in the 180–190 cm${}^{-1}$ range, which is the region where peaks corresponding to crystalline InSb are typically measured; (**b**) spectrum obtained for growth at −1.25 V shows similar defect related peaks as observed in (**a**). There was an absence of any peak in the 180–190 cm${}^{-1}$ range; (**c**) spectrum obtained from nanowires grown at −1.5 V. Well-defined peaks were observed at 149 cm${}^{-1}$, around 120 cm${}^{-1}$ and around 181 cm${}^{-1}$. The zoomed in Raman spectrum image shows two distinct peaks at 180.5 cm${}^{-1}$ and 188.7 cm${}^{-1}$, which is assigned to the c-InSb transverse optical (TO) and longitudinal optical (LO) phonon modes, respectively. In all three cases, the Raman peaks measured in the range from 145–149 cm${}^{-1}$ is attributed to TO-TA modes and is believed to originate from defects in the nanowires, as is the peak around 119.7 cm${}^{-1}$.

Figure 5a–c shows the Raman spectrum obtained from a single or a few InSb nanowires (shown in figure inset). The Raman spectrum for all three deposition potentials is dominated by the peak at about 147–149 cm${}^{-1}$ and a wide peak centered at 120 cm${}^{-1}$. These peaks have been reported by other works [33,38], and are assigned to transverse optical (TO) and transverse acoustic (TA) and 2TA phonons. They have been attributed to defects and also to the amorphous/polycrystalline nature of the nanowires [39,40,41]. The peak at 149 cm${}^{-1}$ has been attributed to a high density of Sb-Sb bonds, typically found in a-InSb [42].

The presence of Sb related defects is most likely related to growth related parameters like the deposition potential and pH of electrolytes. Thus, although the SEM images showed relatively smooth nanowires, the amorphous/polycrystalline nature of the as-grown nanowires was first evident in the Raman spectrum. There was a difference in the Raman spectrum obtained from a single InSb nanowire grown at −1.5 V. The room temperature spectrum reveals peaks of TO and LO phonon modes at 180.5 cm${}^{-1}$ and 188.7 cm${}^{-1}$ respectively, which matches closely with the TO and LO peaks reported in previous studies on InSb nanowires [1,43,44,45,46]. Figure 5b is a zoomed-in view of these two peaks, which are typically reported in c-InSb nanowires.The lack of crystallinity in nanowires grown at very low deposition potentials deserves further investigation. Since the crystalline peaks were only found in nanowires grown at −1.5 V, all further analysis including XRD measurements, HRTEM and electron transport measurements were done on nanowires grown at this potential (−1.5 V).

**Figure 6:**
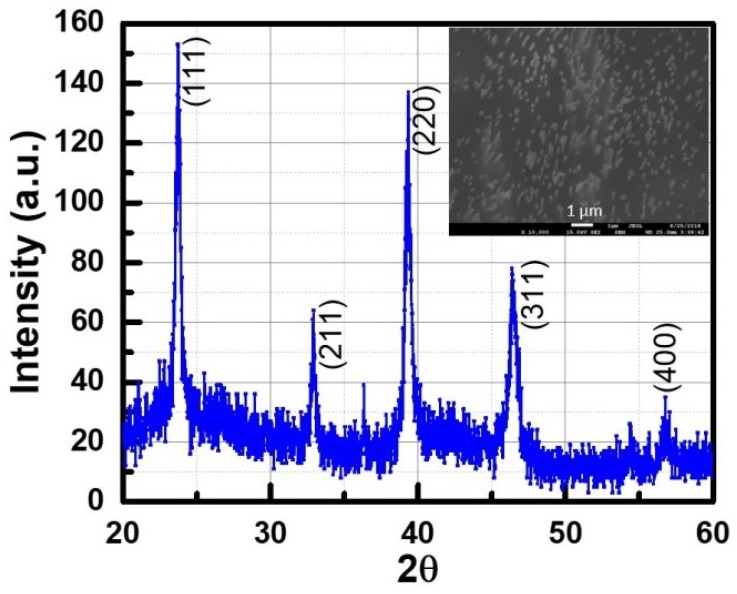
X-ray diffraction (XRD) spectrum of as grown InSb nanowire in polycarbonate template.

To verify the findings from Raman measurements and to further investigate the orientation and crystalline quality of the nanowires, X-ray diffraction studies were performed on the nanowire array grown at −1.5 V. Since it is very difficult to get an XRD pattern from a single nanowire, the XRD pattern was taken from nanowires embedded in the PC membrane. It was first verified that a bare template does not give any XRD signal. Only templates that were filled with nanowires produced an XRD pattern (shown in Figure 6).

The inset of Figure 6 shows the SEM image from where XRD data were collected. The InSb diffraction pattern matched the JCPDS file (00-006-0208) for a cubic zinc blende crystal structure. The sharp peak around 2θ = 23.70${}^{\circ }$ corresponds to (111) crystallographic direction, while the peak at 2θ = 39.36${}^{\circ }$ corresponds to the (220) direction. The reflections ((111), (220), (311), (400)) corresponded to a random crystal structure with no preferred orientation. Another peak is observed at ≈ 33.71${}^{\circ }$, probably due to a thick oxide layer around the nanowires. The presence of this oxide layer is also confirmed by TEM. The lattice constant along different lattice planes was calculated and the average value was estimated to be 0.658 nm, with an average mismatch of about 1.55%.

**Figure 7:**
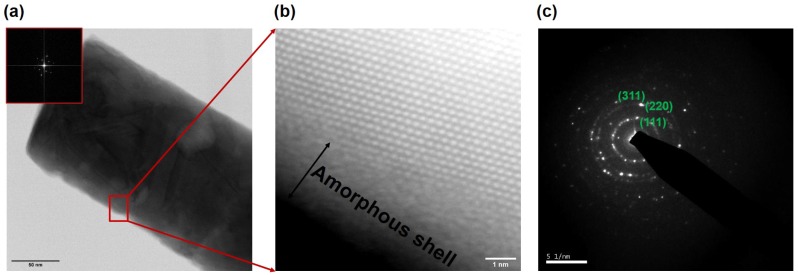
A scanning transmission electron microscope (STEM) images is taken along a single InSb nanowire clearly showing the presence of an amorphous oxide shell: (**a**) Annular bright-field (ABF)-STEM image of a relatively smooth nanowire that shows several crystal grains; the inset shows a fast Fourier transform (FFT) of the image indicating multiple crystallographic orientations; (**b**) magnified high-angle annular dark-field (HAADF)-STEM image of outlined region in (**a**), looking down the (111) direction of one of the larger grains, with a measured distance of 0.25 nm; (**c**) selected area diffraction pattern (SAED) shows indexed rings with discrete spots confirming polycrystalline nature of the nanowire.

In order to probe the microstructures of the as-grown InSb nanowires and obtain information about its crystallographic orientation, structure and surface, results from transmission electron microscopy as well as high-angle annular dark-field (HAADF) scanning transmission electron microscopy (STEM) measurements were analyzed. These studies revealed that two types of nanowires were grown in the template pores: one type was composed of polycrystalline nanowires that under low magnification appeared to have a smooth surface. The second type of nanowire was found to have a rough surface. (Figure 7a) shows an HAADF image of a smooth wire, in which the grain size is on the order of 50 nm (estimation based on what seems to be about 3–5 different grains as seen in the Fast Fourier Transform (FFT) of the image); the inset depicts a FFT of the image confirming its polycrystalline nature. An individual grain oriented to be viewed along its [111] zone axis is observed in the atomic resolution HAADF image in Figure 7b, where the measured distance between atomic columns is 0.28 nm.

The presence of a complex microstructure with randomly oriented nanocrystals in these nanowires was also revealed in the indexed rings in the SAED pattern of Figure 7c that shows diffraction patterns corresponding to the lattice indices of face-centered cubic (fcc) InSb.

**Figure 8:**
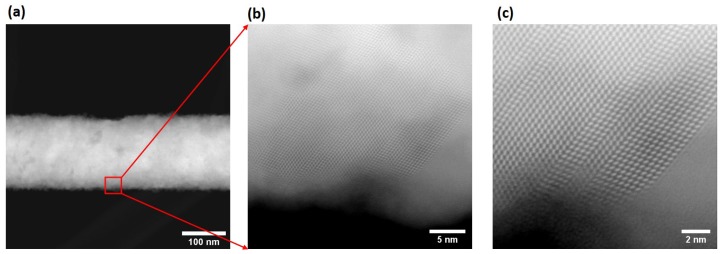
(**a**) HAADF-STEM image taken from a significantly rougher nanowire; (**b**) HAADF image of outlined region in (**a**) showing the presence of crystal defects, which accounts for the observed roughness in the nanowire; (**c**) magnified HAADF image of (**b**) showing twin planes and stacking faults.

An example of a nanowire with a rougher surface can be seen in the HAADF images of Figure 8a–c respectively, where the parallel stripes observed in (b) and (c) indicate the presence of stacking-faults and twinning defects.

A magnified section of the wire from Figure 8a shows a high density of twin defects and stacking faults, the most likely cause for the rough-surface morphology in the as-grown nanowires. The rough nanowires are therefore not a perfect single crystal, but rather one with a dense distribution of stacking faults. Such crystal defects are common in metal nanowires grown in template pores, and has been explained based on low-dimensional growth in spatially confined pores of the template [47]. In both types of nanowires, an amorphous layer was clearly visible on the nanowire surface and its composition as determined by EDX was of In${}_{2}$O${}_{3}$. The presence of an oxide was also seen in the XRD spectum of Figure 6. A model of the zinc blende InSb along the (111) and (220) directions [48] reveals that, along the (111) direction, one can only see the Sb atoms with an atomic separation of around 0.37 nm, validating our observation of similar spacing measured in the HAADF-STEM images of Figure 7. Similarly, it has been reported that, along the (220) direction, the separation between In and Sb atoms is about 0.23 nm, which explains the measured spacing of about 0.25 nm measured in the FFT analysis. The two major lattice spacing measured in the HAADF image ( Figure 8c) was determined to be about 0.37 nm and 0.23 nm corresponding to the (111) and (220) directions, respectively, of zinc blend InSb.

Based on our experimental findings of two distinct nanowire morphologies observed in a single growth cycle, we conclude that different growth modes are at play in their formation. From the growth curves of Figure 2, the layer + island growth mode that is characteristic of the S-K growth mode was implicated, which accounts for the observed polcrsytallinity in the as-grown nanowires.

**Figure 9:**
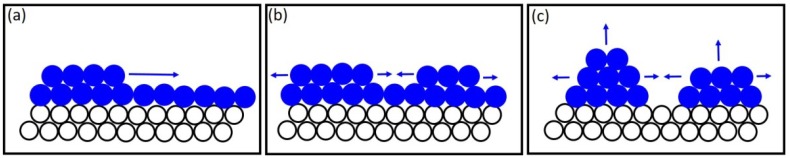
A schematic of the ‘nucleation-coalescence’ growth mechanism, where: (**a**) a few monolayers of InSb grow on the Au electrode; (**b**) islands from over the monloayer; (**c**) coalescence of the islands to form individual crystals/grains.

Another competing mechanism that controls nanowire morphology is the Frank–van der Merwe growth mode, where a 2D nucleus forms and nanowire growth occurs layer by layer. However, in this growth mode, compressive strain in the growing layer imposed by the confined pore environment as well as lattice mismatch between the metal electrode and the growing layer result in crystal growth with a lot of defects. There are several works explaining polycrystallinity in Au [49], Ni [50] nanowires. In these works, it has been proposed that polycrystalline nanowire growth most likely follows the 3D ‘nucleation-coalescence’ mechanism. A schematic of this growth mechanism is shown in Figure 9. The first stage of growth involves the formation of a few angstroms of InSb on top of the metal seed (Au) that exists at the bottom of the template pores (Figure 9a). As growth proceeds, isolated islands form over this crystalline layer and coalesce to form a linked network (Figure 9b,c). The growth mechanism is sensitive to the growth environment which includes the condition of the template pore interior, the nature of the electrodes at which nucleation begins, the electrolyte and the deposition potential. It has been reported [50] that in, electrochemical deposition, there exists a critical dimension $d_{c}$ beyond which new crystals form. For single crystal growth, the value of $d_{c}$ should be large and the most appropriate way to achieve this is to maintain a low deposition potential. It is worth noting that electrochemical growth of nanowires in template pores is a complicated process and the nanowire quality is affected by the movement of ions in the pores, process of diffusion, reaction and adsorption to the metal seed at bottom of pore. All of these factors are related to deposition conditions and influence the growth modes, which in turn are influenced by surface energy of the growing layers. For a 2D-nucleus growth, the critical size of a 2D nucleus $d_{c}$ is inversely proportional to the square of the overpotential [51], whereas, for a 3D-like nucleus, $d_{c}$ is inversely proportional to the cube of the overpotential. At the higher deposition potential of −1.5 V, the surface diffusion of In and Sb atoms favors their aggregation resulting in nucleation and growth of several small 3D crystals that grow simultaneously and independently of each other during the first few minutes of growth. The polycrystalline nanowires showed relatively large grain sizes (about 50 nm), which implies that single crystal growth of semiconductors like InSb in template pores is affected by several factors unlike what has been observed in growth of metallic nanowires.

On the other hand, if the growth environment in the membrane pores is such that it inhibits the formation of several 3D nuclei, and instead a 2D nucleus forms, then it favors single crystal growth. Nanowire growth in this case follows the two-dimensional (2D) nucleation/growth mechanism. Since growth occurs in a confined pore, lateral stresses develop in the growing crystal as a result of the confined growth process. This results in the growth of InSb nanowires with high defect density. The process effectively forces the nanowires to crystallize in the cylindrical geometry of the membrane pores, resulting in formation of stacking faults as the crystal growth rate in different directions were disturbed. This results in formation of 111 stacking faults and twin defects of the nanowire crystal lattice. The high density of defects is responsible for the rough morphology of some of the InSb nanowires.

**Figure 10:**
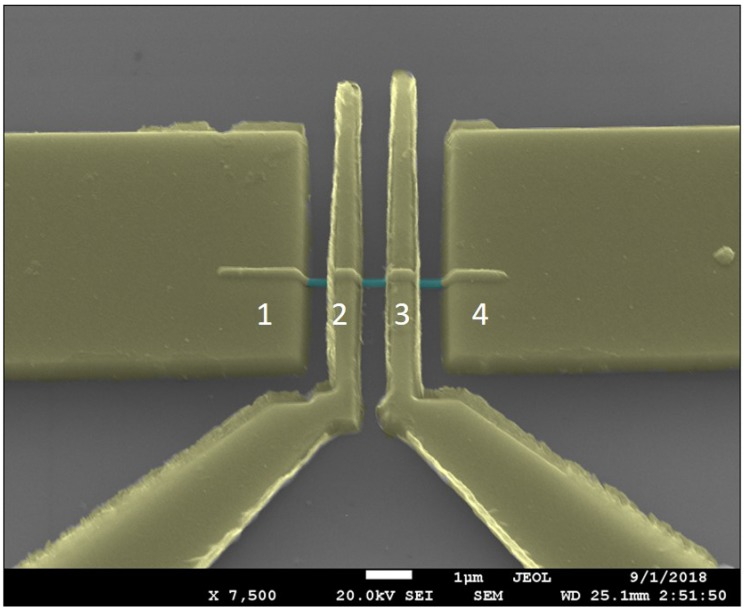
SEM image of single InSb nanowire contacted by four Ni electrodes. The nanowire was placed on a 200 nm thick silicon dioxide ( SiO${}_{2}$) grown over a highly doped $p^{+}$ Si substrate. The channel length between each electrode is about 1.5 μm. The metal electrodes are Cr (5 nm)/Ni (200 nm). Cr enables better adhesion of Ni film on the SiO${}_{2}$ layer.

To assess the electronic quality of the nanowires, current–voltage measurements were made on a single InSb nanowire that was contacted by Ni electrodes, which is shown in Figure 10.

**Figure 11:**
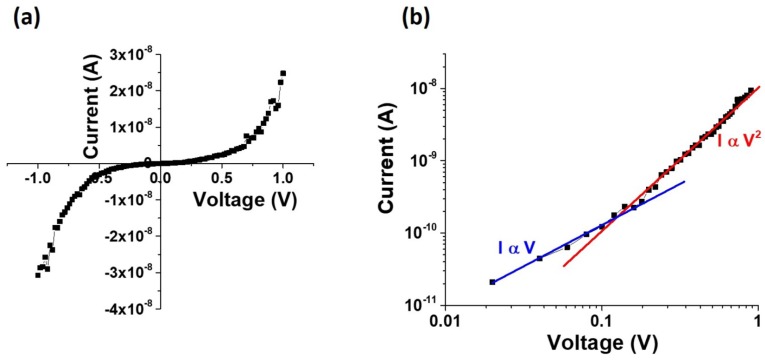
Current–voltage measurements on a single as-grown Insb nanowire. The nanowire diameter and electrode spacing are 190 nm and 1.5 μm, respectively. (**a**) symmetric nonlinear I–V curve on linear scale. The curves are linear (ohmic) at small bias and transforms at a high bias; (**b**) I–V curves plotted on a logarithmic scale, showing a crossover to space charge limited current (SCLC) at $V_{x}$ = 0.14 V.

Figure 11 shows the experimental I–V curves on a linear and a double-logarithmic scale. As seen in this plot, the fairly symmetric nonlinear I–V plot shows two distinct regions: (i) at small bias I ∝ V; (ii) at larger bias, I ∝ V${}^{2}$. This behavior is characteristic of space charge limited current (SCLC) that is typically observed in very resistive materials with ohmic contacts [51]. The contacted cylindrical nanowires have a contacted length of 3.0 μm, which is much greater than the nanowire radius of 100 nm (L >> R).

This results in reduced electrostatic screening effects, which implies that charge injection effects by the contacts will be much higher on these devices as compared to a bulk structure. Since the electrical contacts to the two ends of the nanowire are symmetric, it leads to fairly symmetric I–V plots for positive and negative bias. Since we do not observe any exponential trend in the lot, we do not consider Schottky barriers at the contacts. We therefore believe that the current changes from ohmic at low bias to SCLC at high bias. Using a four-terminal device configuration, it was also verified that the contact resistance was much lower than the wire resistance. In the low bias regime, the as-grown nanowire has a resistance of the order of $10^{9}$Ω, while the contact resistance is of the order of kΩ. The high nanowire resistance is to be expected at low bias, since, in this bias regime, the injected electron concentration is low compared to the equilibrium electron concentration and the magnitude of the current follows Ohm’s law where I ∝ V. The cross-over point in the I–V characteristics occurs at about 0.14 V, which is most likely the point at which the injected electron concentration exceeds the equilibrium electron concentration. Since the contact dimensions are much larger than that of the nanowire, the behavior of the current variation with bias in this regime is expressed by the Mott–Gurney law in which I ∝ V${}^{2}$. From the I–V curve of Figure 11, the cross-over voltage of $V_{x}$ = 0.14 V is used to make a rough estimate of the free carrier concentration in the nanowire, using Equation [51]

(1) $$n=\frac{\epsilon_{0}V_{x}}{(e\pi R^{2}ln\left(L/R\right)}\prime$$

where $\epsilon_{0}$ is the permittivity of free space and L and R represent the nanowire dimensions. Using this model, the free carrier concentration is estimated to be of the order of $10^{14}$ cm${}^{-3}$. This value is about an order of magnitude lower than that reported for undoped bulk c-InSb. This discrepancy could be attributed to the fact that, in using Equation (1), we have assumed that the symmetry in I–V characteristics is indicative of a symmetric electric filed created by the contacts around the nanowire. However, with the polycrystalline nanowires, the conduction will be non-uniform and the presence of defects like stacking faults could further impact the conduction mechanisms. SCLC is typically observed in undoped nanowires with low free carrier concentration where the ohmic current is controlled by charges from bulk contacts diffusing through the nanowire, rather than from impurities or dopants. The effect of polycrystallinity on the conduction mechanism and hence on the SCLC is a topic for further study.

## 4. Conclusions

Polycrystalline and crystalline InSb nanowires with high defect density were synthesized at room temperature using a direct current (DC) electrodeposition process in porous polycarbonate membrane. The structural quality of the nanowires was assessed by high resolution transmission electron microscopy, Raman spectroscopy and X-ray diffraction studies. An important parameter that determines the structural quality of the nanowires is the critical grain size whose value changes with deposition potential. The Stranski–Krastanov growth mode best explains the growth of polycrystalline InSb nanowires by a 3D ‘nucleation-coalescence’ mechanism. On the other hand, the growth of crystalline nanowires follows the Frank–van der Merwe growth mechanism that follows a 2D nucleation epitaxial growth. On account of reduced carrier screening in the nanowire geometry, the phenomenon of space charge limited current was observed due to increased charge injection at high bias fields. At low bias, current follows the traditional ohmic behavior. The cross-over voltage was used to determine an equilibrium carrier concentration of $10^{14}$ cm${}^{-3}$.

## References

[1] Zhang X., Hao Y., Meng G., Zhang L. (2005). Fabrication of Highly Ordered InSb Nanowire Arrays by Electrodeposition in Porous Anodic Alumina Membranes. J. Electrochem. Soc..

[2] Heyns M., Tsai W. (2009). Ultimate Scaling of CMOS Logic Devices with Ge and III–V Materials. MRS Bull..

[3] Vogel A.T., de Boor J., Wittemann J.V., Mensah S.L., Werner P., Schmidt V. (2011). Fabrication of High-Quality InSb Nanowire Arrays by Chemical Beam Epitaxy. Cryst. Growth Des..

[4] Nilsson H.A., Caroff P., Thelander C., Larsson M., Wagner J.B., Wernersson L.-E., Samuelson L., Xu H.Q. (2009). Giant, Level-Dependent *g* Factors in InSb Nanowire Quantum Dots. Nano Lett..

[5] Vurgaftman I., Meyer J.R., Ram-Mohan L.R. (2001). Band parameters for III–V compound semiconductors and their alloys. J. Appl. Phys..

[6] Radosavljevic M., Ashley T., Andreev A., Coomber S.D., Dewey G., Emeny M.T., Fearn M., Hayes D.G., Hilton K.P., Hudait M.K. High-performance 40nm gate length InSb p-channel compressively strained quantum well field effect transistors for low-power (VCC=0.5V) logic applications. Proceedings of the 2008 IEEE International Electron Devices Meeting.

[7] Del Alamo J.A. (2011). Nanometre-scale electronics with III–V compound semiconductors. Nature.

[8] Das S.R., Delker C.J., Zakharov D., Chen Y.P., Sands T.D., Janes D.B. (2011). Room temperature device performance of electrodeposited InSb nanowire field effect transistors. Appl. Phys. Lett..

[9] Zhao Y., Candebat D., Delker C., Zi Y., Janes D., Appenzeller J., Yang C. (2012). Understanding the Impact of Schottky Barriers on the Performance of Narrow Bandgap Nanowire Field Effect Transistors. Nano Lett..

[10] Ashley T., Buckle L., Datta S., Emeny M.T., Hayes D.G., Hilton K.P., Jefferies R., Martin T., Phillips T.J., Wallis D.J. (2007). Heterogeneous InSb quantum well transistors on silicon for ultra-high speed, low power logic applications. Electron. Lett..

[11] Chen H., Sun X., Lai K.W.C., Meyyappan M., Xi N. Meyyappan and N. Xi, Infrared detection using an InSb nanowire. Proceedings of the 2009 IEEE Nanotechnology Materials and Devices Conference.

[12] Chen H., Lai K.W.C., Sun X., Xi N., Meyyappan M. (2012). Chapter 13—Indium Antimonide (InSb) Nanowire- Based Photodetectors. Nano Optoelectronic Sensors and Devices.

[13] Rogalski A. (2003). Infrared detectors: Status and trends. Prog. Quantum Electron..

[14] Zhou F., Seol J.H., Moore A.L., Shi L., Ye Q.L., Scheffler R. (2006). One-dimensional electron transport and thermopower in an individual InSb nanowire. J. Phys. Condens. Matter.

[15] Zhou F., Moore A.L., Pettes M.T., Lee Y., Seol J.H., Ye Q.L., Rabenberg L., Shi L. (2010). Effect of growth base pressure on the thermoelectric properties of indium antimonide nanowires. J. Phys. D Appl. Phys..

[16] Chen Z.-G., Han G., Yang L., Cheng L., Zou J. (2012). Nanostructured thermoelectric materials: Current research and future challenge. Prog. Nat. Sci. Mater. Int..

[17] Weathers A., Shi L. (2013). Thermal transport measurement techniques for nanowires and nanotubes. Annu. Rev. Heat Transf..

[18] Mingo N. (2004). Thermoelectric figure of merit and maximum power factor in III–V semiconductor nanowires. Appl. Phys. Lett..

[19] Solin S.A., Thio T., Hines D.R., Heremans J.J. (2000). Enhanced Room-Temperature Geometric Magnetoresistance in Inhomogeneous Narrow-Gap Semiconductors. Science.

[20] Wu Y., Yang P. (2001). Direct Observation of Vapor-Liquid-Solid Nanowire Growth. J. Am. Chem. Soc..

[21] Li S., Kang N., Fan D.X., Wang L.B., Huang Y.Q., Caroff P., Xu H.Q. (2016). Coherent Charge Transport in Ballistic InSb Nanowire Josephson Junctions. Sci. Rep..

[22] Cao G., Liu D. (2008). Template-based synthesis of nanorod, nanowire, and nanotube arrays. Adv. Colloid Interface Sci..

[23] Lai M., Riley D.J. (2008). Templated electrosynthesis of nanomaterials and porous structures. J. Colloid Interface Sci..

[24] Martín-González M., Snyder G.J., Prieto A.L., Gronsky R., Sands T., Stacy A.M. (2003). Direct Electrodeposition of Highly Dense 50 nm Bi_2_Te_3_-ySey Nanowire Arrays. Nano Lett..

[25] Liu C., Gillette E.I., Chen X., Pearse A.J., Kozen A.C., Schroeder M.A., Gregorczyk K.E., Lee S.B., Rubloff G.W. (2014). An all-in-one nanopore battery array. Nat. Nanotechnol..

[26] Hochbaum A.I., Chen R., Delgado R.D., Liang W., Garnett E.C., Najarian M., Majumdar A., Yang P. (2008). Enhanced thermoelectric performance of rough silicon nanowires. Nature.

[27] Chan C.K., Peng H., Liu G., McIlwrath K., Zhang X.F., Huggins R.A., Cui Y. (2008). High-performance lithium battery anodes using silicon nanowires. Nat. Nanotechnol..

[28] Xu S., Qin Y., Xu C., Wei Y., Yang R., Wang Z.L. (2010). Self-powered nanowire devices. Nat. Nanotechnol..

[29] MGudiksen S., Lauhon L.J., Wang J., Smith D.C., Lieber C.M. (2002). Growth of nanowire superlattice structures for nanoscale photonics and electronics. Nature.

[30] Duan X., Fu T.-M., Liu J., Lieber C.M. (2013). Nanoelectronics-biology frontier: From nanoscopic probes for action potential recording in live cells to three-dimensional cyborg tissues. Nano Today.

[31] Cui Y., Lieber C.M. (2001). Functional Nanoscale Electronic Devices Assembled Using Silicon Nanowire Building Blocks. Science.

[32] Shin S., Al-Housseiny T.T., Kim B.S., Cho H.H., Stone H.A. (2014). The Race of Nanowires: Morphological Instabilities and a Control Strategy. Nano Lett..

[33] Das S.R., Akatay C., Mohammad A., Khan M.R., Maeda K., Deacon R.S., Ishibashi K., Chen Y.P., Sands T.D., Alam M.A. (2014). Electrodeposition of InSb branched nanowires: Controlled growth with structurally tailored properties. J. Appl. Phys..

[34] Bard A.J., Stratmann M., Licht S. (2002). Semiconductor Electrodes and Photoelectrochemistry, Encyclopedia of Electrochemistry.

[35] Lincot D. (2005). Electrodeposition of semiconductors. Thin Solid Films.

[36] Zech N., Podlaha E.J., Landolt D. (1999). Anomalous Codeposition of Iron Group Metals: I. Experimental Results. J. Electrochem. Soc..

[37] Ortega J., Herrer J. (1989). Preparation of In X (X = P, As, Sb) Thin Films by Electrochemical Methods. J. Electrochem. Soc..

[38] Wada N., Takayama H., Morohashi S. Abstract No. L9.013. Proceedings of the APS March Meeting 55.

[39] Demishev S., Kosichkin Y.V., Lyapin A., Mel’Nik N., Nekhaev D., Sluchanko N., Turok A. (1993). Raman scattering in amorphous gallium antimonide. Eksp. Teor. Fiz..

[40] Wihl M., Cardona M., Tauc J. (1972). Raman scattering in amorphous Ge and **III**–**V** compounds. J. Non Cryst. Solids.

[41] Lannin J.S. (1972). Low frequency Raman scattering in amorphous materials: A-Ge, a-InSb, and a-Ge_0.5_Sn_0.5_. Solid State Commun..

[42] Algarni Z., Singh A., Philipose U. (2018). Synthesis of Amorphous InSb Nanowires and a Study of the Effects of Laser Radiation and Thermal Annealing on Nanowire Crystallinity. Nanomaterials.

[43] Kiefer W., Richter W., Cardona M. (1975). Second-order Raman scattering in InSb. Phys. Rev. B.

[44] Wang Y., Chi J., Banerjee K., Grützmacher D., Schäpers T., Lu J.G. (2011). Field effect transistor based on single crystalline InSb nanowire. J. Mater. Chem..

[45] Pinczuk A., Burstein E. (1968). Raman Scattering from InSb Surfaces at Photon Energies Near the *E*_1_ Energy Gap. Phys. Rev. Lett..

[46] Wagner V., Drews D., Esser N., Zahn D.R.T., Geurts J., Richter W. (1994). Raman monitoring of semiconductor growth. J. Appl. Phys..

[47] (2012). Maria Eugenia Toimil-Molares, Characterization and properties of micro- and nanowires of controlled size, composition, and geometry fabricated by electrodeposition and ion-track technology. Beilstein Nanotechnol..

[48] Pandya G., Kordesch M.E. (2015). Characterization of InSb Nanoparticles Synthesized Using Inert Gas Condensation. Nanoscale Res. Lett..

[49] Liu J., Duan J.L., Toimil-Molares M.E., Karim S., Cornelius T.W., Dobrev D., Yao H.J., Sun Y.M., Hou M.D., Mo D. (2006). Electrochemical fabrication of single-crystalline and polycrystalline Au nanowires: The influence of deposition parameters. Nanotechnology.

[50] Tian M., Wang J., Kurtz J., Mallouk T.E., Chan M.H.W. (2003). Electrochemical Growth of Single-Crystal Metal Nanowires via a Two-Dimensional Nucleation and Growth Mechanism. Nano. Lett..

[51] Alagha S., Shik A., Ruda H.E., Saveliev I., Kavanagh K.L., Watkins S.P. (2017). Space-charge-limited current in nanowires. J. Appl. Phys..

